# The Bidirectional Association Between Bullous Pemphigoid and Psoriasis: A Population-Based Cohort Study

**DOI:** 10.3389/fmed.2020.00511

**Published:** 2020-08-31

**Authors:** Khalaf Kridin, Ralf J. Ludwig, Yochai Schonmann, Giovanni Damiani, Arnon D. Cohen

**Affiliations:** ^1^Department of Experimental Dermatology, Lubeck Institute, University of Lübeck, Lübeck, Germany; ^2^Department of Quality Measurements and Research, Clalit Health Services, Tel Aviv, Israel; ^3^Clinical Dermatology, IRCCS Istituto Ortopedico Galeazzi, Milan, Italy; ^4^Siaal Research Center for Family Medicine and Primary Care, Faculty of Health Sciences, Ben-Gurion University of the Negev, Beer Sheva, Israel; ^5^Department of Biomedical, Surgical and Dental Sciences, University of Milan, Milan, Italy

**Keywords:** bullous pemphigoid, psoriasis, cohort study, case-control study, bidirectional

## Abstract

The risk of developing psoriasis during the course of bullous pemphigoid (BP) is yet to be investigated. We aimed to assess the risk of psoriasis among patients with BP and the risk of BP in individuals with a history of psoriasis. A population-based retrospective cohort study was conducted comparing BP patients (*n* = 3,924) with age-, sex-, and ethnicity-matched control subjects (*n* = 19,280) with regard to incident cases of psoriasis. A case-control design was additionally followed to estimate the risk of BP in those with a preceding diagnosis of psoriasis. Adjusted hazard ratios (HRs) and adjusted odds ratios (ORs) were estimated by Cox regression and logistic regression, respectively. The incidence of psoriasis was 1.78 (95% CI, 1.25–2.48) and 0.67 (95%CI, 0.53–0.83) per 1,000 person-years among patients with BP and controls, respectively. Patients with BP were 2.6-fold more likely to develop psoriasis (HR, 2.60; 95%CI, 1.59–4.27) compared to controls. Furthermore, the prevalence of preexisting psoriasis was higher in patients with BP than in control subjects (1.7 vs. 1.1%, respectively; *P* < 0.001). A history of psoriasis was associated with a 50% increase in the risk of BP (OR, 1.53; 95%CI, 1.17–2.02). Patients with a dual diagnosis of BP and psoriasis were younger, had higher prevalence of smoking and hypertension, and were treated more frequently with prolonged systemic and topical corticosteroids when compared to the remaining patients with BP. To conclude, a bidirectional association exists between BP and psoriasis. Awareness of this association may be of great importance for physicians managing patients with BP and psoriasis.

## Introduction

Bullous pemphigoid (BP) is the most prevalent subepidermal autoimmune blistering disease (AIBD) worldwide (1). Recent evidence accumulated to suggest that BP confers an increasing inpatient burden (2) owing to its growing incidence (3), its increased associated mortality (4, 5), and high healthcare costs (6). This tissue-specific autoimmune disease arises from the production of IgG autoantibodies targeting the hemidesmosomal proteins BP180 and BP230 (7) and typically afflicts the elderly population (1).

Psoriasis is a chronic papulosquamous dermatosis which affects 2–4% of the Western world population (8, 9). It is caused by dysregulation of the immune system and manifests clinically with scaly erythematous plaques. While it is a held belief that inflammation driven by the tumor necrosis factor-α/interleukin-23/interleukin-17 axis is the key pathomechanism of psoriasis, it also possesses an autoimmune facet that manifests as autoreactive T cells (10). The association of psoriasis with several autoimmune diseases and the detection of autoreactive T cells in psoriasis patients lends weight to this concept (10, 11).

Psoriasis seems to be overrepresented among patients with BP. Data across four cross-sectional and case-control studies were recently synthetized to reveal that the prevalence of psoriasis was 2.5-times increased in patients with BP (12). However, the risk of developing psoriasis during the course of BP has not been investigated to-date. The question of whether patients with coexistent BP and psoriasis possess distinct clinical characteristics is yet to be decisively answered.

The aim of the current study was to estimate the bidirectional association between BP and psoriasis. We herein sought to evaluate the prevalence of preexisting psoriasis among patients newly diagnosed with BP, as well as their subsequent risk of developing psoriasis. The last endpoint of the current study was to characterize patients with coexistent BP and psoriasis as compared to the remaining patients with BP.

## Methods

### Study Design and Dataset

The current study was performed to investigate the bidirectional association between BP and psoriasis using one of the large cohorts of patients with BP in the literature. To outline the risk of developing psoriasis during the course of BP, a cohort study deign was adopted to follow patients with BP and estimate the incidence of psoriasis. To evaluate the risk of having BP in individuals with a preceding history of psoriasis, a case-control study design was followed to disclose the prevalence of preceding psoriasis (exposure) in patients with subsequent BP (outcome). “The rare disease assumption” hypothesizes that estimates produced by case-control studies investigating rare diseases approximates that produced by cohort studies (13). Thus, the case-control study utilized in the current study enabled to evaluate the risk of BP in subjects with a history of psoriasis.

The current study was grounded on the computerized dataset of Clalit Health Services (CHS), the main healthcare provider in Israel which provides a wide array of private and public healthcare services for 4,927,000 enrollees as of October 2018. CHS is typified by a prominent inclusiveness since it retrieves data from a multitude of sources covering general community clinics, both primary care and referral center settings, and both ambulatory and hospitalized care settings. The loss to follow-up is minimal and access to CHS services is free, rendering this dataset highly compatible with reliable epidemiological studies (14).

### Study Population and Definition of Main Variables

The computerized dataset of CHS was systematically checked for incident cases with a diagnostic code compatible with BP between the years 2002 and 2019. Eligibility in the study was determined only if patients fulfilled at least one of the following: (i) a documented diagnosis of BP registered at least twice by a community-based board-certified dermatologist, or (ii) diagnosis of BP in discharge letters of patients admitted to dermatological wards. The diagnosis of psoriasis was based on its documentation in the medical record by a board-certified dermatologist.

The control group was comprised of up to 5 individuals per each case of BP. Controls were matched based on sex, age, and ethnicity. Prior to their recruitment, controls were ascertained to be alive and to contribute longitudinal data to the CHS.

### Covariates and Sensitivity Analyses

The outcome measures of the study were adjusted for comorbid conditions as estimated by the Charlson Comorbidity Index (CCI), an epidemiological score enabling to assess the extent and severity of comorbidities (15). The latter is methodologically valid in large data analyses and was found to reliably predict mortality. Since both investigated diseases are implicated with an increased prevalence of metabolic outcomes (16, 17), we have additionally adjusted for body mass index (BMI), the presence of diabetes mellitus, hypertension, dyslipidemia, and smoking. These variables were retrieved from the chronic registry of CHS.

To substantiate the validity of our findings, we performed two sensitivity analyses alongside the general analysis; (i) to increase the validity of the diagnosis of BP, only patients who were prescribed BP-related medications were included. This includes systemic corticosteroids for more than 6 months, topical corticosteroids for more than 6 months, and one of the adjuvant immunosuppressive or immunomodulatory agents (azathioprine, mycophenolate mofetil, methotrexate, cyclophosphamide, dapsone, doxycycline, rituximab, plasmapheresis, intravenous immunoglobulins); (ii) to refute the presence of major ascertainment bias in the case-control study, cases of psoriasis diagnosed up to 2 years prior to the study initiation (date diagnosis of cases and recruitment of controls) were omitted.

### Statistical Analysis

Baseline characteristics were described by means and standard deviations (SD)s for continuous variables, whilst categorical values were indicated by percentages. The comparison of different variables between cases and controls was performed using the chi-square test and *t*-test for categorical and continuous variables, respectively.

In the cohort study design, incidence rates of psoriasis were calculated for both BP patients and controls and expressed as the number of events per 1,000 person-years. The incidence of psoriasis during follow-up was calculated only for individuals without a history of psoriasis before study initiation. Hazard ratios (HR)s for the risk of incident psoriasis were obtained by the use of the Cox regression model. The cumulative incidence of psoriasis was compared between BP and control groups using a stratified log-rank test.

In the case-control study design, logistic regression was used to calculate odds ratios (ORs) and 95% confidence intervals (CI)s to compare cases and controls regarding the presence of preceding psoriasis. The association was calculated based on individuals who developed BP after the diagnosis of psoriasis, given that a temporal relationship exists between exposure and outcome.

While investigating the epidemiological and clinical characteristics of patients with BP and psoriasis relative to those with isolated BP, all patients with both diagnoses were included, regardless of the sequence of their appearance. Two-tailed *P* < 0.05 were considered as statistically significant. All statistical analyses were performed using SPSS software, version 25 (SPSS, Armonk, NY: IBM Corp).

## Results

### Characteristics of The Study Population

The study population included 3,924 patients with BP and 19,280 matched control subjects. The mean (SD) age at the diagnosis of patients was 76.7 (14.3) years, 2,257 (57.5%) patients were females, and 3,752 (95.6%) patients were of Jewish ethnicity. The age at recruitment, sex distribution, and ethnic background was not significantly different among control participants (Table 1). The prevalence of smoking and the average BMI were comparable in patients with BP and controls (Table 1). The mean (SD) CCI score was greater in cases than in controls [3.4 (2.4) vs. 2.9 (2.3), respectively; *P* < 0.001]. The demographic and clinical features of the study participants are detailed in Table 1.

**Table 1:** Descriptive characteristics of the study population.

**Characteristic**	**Patients with bullous pemphigoid (*N =* 3,924)**	**Controls (*N =* 19,280)**	***P*-value**
**Age, years**			
Mean (SD)	76.7 (14.3)	76.3 (14.3)	0.904
Median (range)	79.9 (0.4–104.4)	79.5 (0.7–103.8)	
Male sex, *N* (%)	1,667 (42.5%)	8,168 (42.4%)	0.908
**Ethnicity**, ***N*** **(%)**			
Jews	3,752 (95.6%)	18,397 (95.4%)	0.584
Arabs	171 (4.4%)	868 (4.5%)	
**BMI, mg/kg**^**2**^			
Mean (SD)	27.9 (6.1)	27.9 (8.4)	1.000
Smoking, N (%)	1,148 (29.3%)	5,771 (29.9%)	0.454
**Charlson comorbidity score**			
Mean score (SD)	3.4 (2.4)	2.9 (2.3)	**<0.001**
None (0)	468 (11.9%)	3,376 (17.5%)	**<0.001**
Moderate (1–2)	1,113 (28.4%)	6,177 (32.0%)	**<0.001**
Severe (≥3)	2,343 (59.7%)	9,727 (50.5%)	**<0.001**

*BP, bullous pemphigoid; N, Number; SD, standard deviation; BMI, body mass index. All p-value in bold are statistically significant*.

### The Risk of Developing Psoriasis Among Patients With Bullous Pemphigoid

To address this question, we performed a retrospective cohort study tracking patients with BP for the development of psoriasis. The total follow-up time was 18,500.5 person-years for patients with BP and 110,422.2 person-years for controls. During the follow-up duration, 33 cases of new-onset psoriasis occurred among patients with BP and 74 similar cases among control subjects. Altogether, the incidence rate of psoriasis was 1.78 (95% CI, 1.25–2.48) and 0.67 (95% CI, 0.53–0.84) per 1,000 person-years among patients with BP and controls, respectively (Table 2).

**Table 2:** Incidence rates and hazard ratio of new-onset psoriasis among patients with bullous pemphigoid (cohort study design).

	**Patients with bullous pemphigoid**	**Controls**
Follow-up time, PY	18,500.5	110,422.2
Median follow-up time, years (range)	3.53 (0.00–17.80)	4.88 (0.00–18.01)
Number of events	33	74
Incidence rate/1,000 PY	1.78	0.67
95% CI	1.25–2.48	0.53–0.84
	**HR (95% CI)**	***P*****-value**
Crude	**2.65 (1.75–3.99)**	**<0.001**
Adjusted^*^	**2.60 (1.59–4.27)**	**<0.001**
Sensitivity analysis^**^		
Crude	**2.32 (1.54–3.50)**	**<0.001**
Adjusted^*^	**2.41 (1.46–3.97)**	**0.001**

*HR, hazard ratio; CI, confidence interval; PY, person-year*.

* *Following the adjustment for age, sex, ethnicity, socioeconomic status, diabetes mellitus, body mass index, hypertension, and dyslipidemia*.

** *Sensitivity analysis included only bullous pemphigoid patients under prolonged “bullous pemphigoid -related treatments.” **Bold:** significant value*.

**Figure 1:**
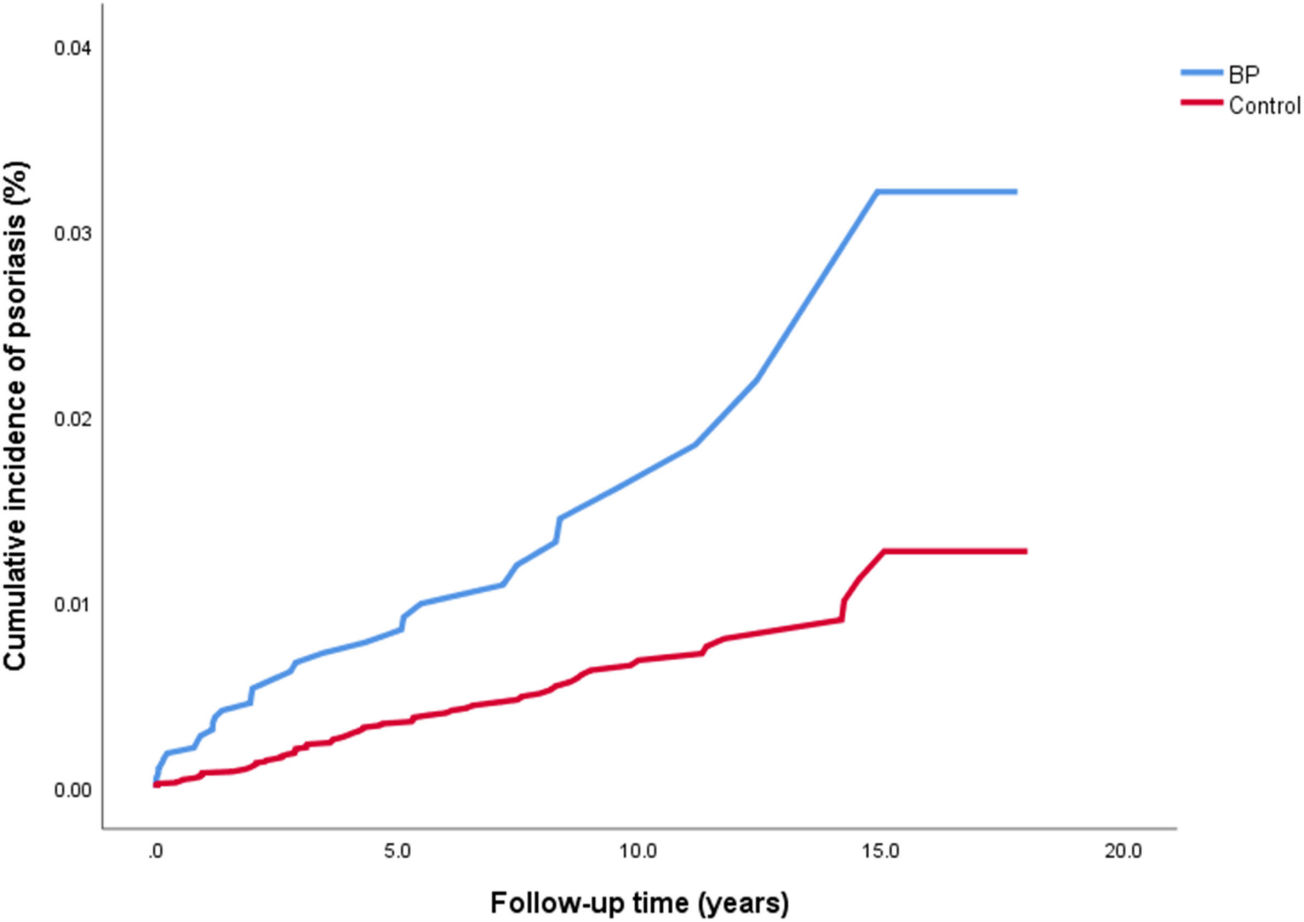
The cumulative incidence of psoriasis among patients with BP.

The crude risk of developing incident psoriasis was 2.7-fold higher among patients with BP (HR, 2.65; 95% CI, 1.75–3.99; *P* < 0.001; Figure 1). The risk of new-onset psoriasis was increased among male (HR, 2.88; 95% CI, 1.64–5.05; *P* < 0.001) and female (HR, 2.43; 95% CI, 1.33–4.44; *P* = 0.004) patients with BP. After controlling for confounding factors like demographic variables, diabetes mellitus, hypertension, BMI, and dyslipidemia, BP emerged as an independent significant risk factor of psoriasis (adjusted HR, 2.60; 95% CI, 1.59–4.27; *P* < 0.001; Table 2).

We then carried out a sensitivity analysis which included only BP patients managed by BP-related medications. The crude risk (HR, 2.32; 95% CI, 1.54–3.50; *P* < 0.001) and the multivariate risk (adjusted HR, 2.41; 95% CI, 1.46–3.97; *P* = 0.001) of psoriasis were slightly attenuated but retained their statistical significance (Table 2).

### The Risk of Developing BP With a Preceding Diagnosis of Psoriasis

The question of whether psoriasis predisposes individuals to develop BP was answered by a case-control study estimating the association between a preceding diagnosis of psoriasis (exposure) and subsequent diagnosis of BP (outcome). The prevalence of preexisting psoriasis was higher among patients with BP relative to control subjects (1.7 vs. 1.1%, respectively; *P* = 0.002). That is, a 1.5-fold increase in the risk of BP was observed in those with a history of psoriasis (OR, 1.53; 95% CI, 1.17–2.02). When stratified by age, sex, and ethnicity, psoriasis was found to predict the diagnosis of BP in patients aged between 71 and 80 years (OR, 1.85; 95% CI, 1.18–2.91), male patients (OR, 1.61; 95% CI, 1.10–2.36), and individuals of both Jewish (OR, 1.47; 95% CI, 1.11–1.95) and Arab ethnicity (OR, 3.25; 95% CI, 1.05–10.06; Table 3).

**Table 3:** The risk of bullous pemphigoid in patients with a preceding diagnosis of psoriasis stratified by age, sex, and ethnicity (case-control study design).

**Subgroup**	**Psoriasis in**	**Psoriasis in**	**OR (95%CI)**	**Univariate**
	**patients with**	**controls**		***P*-value**
	BP *n* (%)^*^	*n* (%)^*^		
**All**	68 (1.7%)	220 (1.1%)	**1.53 (1.17–2.02)**	**0.002**
**Age, years**				
<70	14 (1.6%)	46 (1.0%)	1.58 (0.87–2.89)	0.134
71–80	26 (2.4%)	72 (1.3%)	**1.85 (1.18–2.91)**	**0.007**
≥80	28 (1.4%)	102 (1.1%)	1.31 (0.86–1.99)	0.205
**Sex**				
Male	36 (2.2%)	111 (1.4%)	**1.61 (1.10–2.36)**	**0.013**
Female	32 (1.4%)	109 (1.0%)	1.46 (0.98–2.17)	0.062
**Ethnicity**				
Jews	63 (1.7%)	212 (1.2%)	**1.47 (1.11–1.95)**	**0.007**
Arabs	5 (2.9%)	8 (0.9%)	**3.25 (1.05–10.06)**	**0.031**

* *The prevalence of psoriasis in cases when psoriasis preceded BP (in cases) or preceded recruitment (in controls)*.

*OR, odds ratio; n, Number; CI, confidence interval*.

***Bold:** significant value*.

We then performed two sensitivity analyses. The first of which included only patients with BP managed by BP-related mediations and found a similar estimate (OR, 1.58; 95% CI, 1.19–2.07). The second sensitivity analysis excluded patients diagnosed with psoriasis up to 2 years prior to BP and found that the association was not altered substantially (OR, 1.54; 95% CI, 1.16–2.05; Table 4).

**Table 4:** Sensitivity analyses and multiple logistic regression analysis of the association between psoriasis and the later development of BP (case-control study design).

	**OR**	**95% CI**	***P*-value**
**Sensitivity analyses**			
Sensitivity analysis 1^a^	1.58	1.19–2.07	**0.001**
Sensitivity analysis 2^b^	1.54	1.16–2.05	**0.003**
**Regression model**			
Unadjusted model	1.53	1.17–2.02	**0.002**
Model 1^c^	1.51	1.14–2.00	**0.004**
Model 2^d^	1.53	1.14–2.06	**0.005**

a *including only cases and control diagnosed with psoriasis more than 2 years prior to the initiation of the study (patients diagnosed with psoriasis up to 2 years before the diagnosis of BP or the recruitment of controls were excluded)*.

b *including only cases managed by BP-related medications*.

c *adjusted for age, sex, ethnicity, and socioeconomic status*.

d *adjusted additionally for diabetes mellitus, body mass index, hypertension, and dyslipidemia*.

*CI, confidence interval*.

***Bold:** significant values*.

The association persisted significantly after adjusting for demographic variables (model 1; OR, 1.51; 95% CI, 1.14–2.00) as well as for demographic variables alongside diabetes mellitus, BMI, hypertension, and dyslipidemia (model 2; OR, 1.53; 95% CI, 1.14–2.06; Table 4).

### The Latency Between the Appearance of The Two Investigated Conditions

Of the 110 patients with coexistent BP and psoriasis, BP followed psoriasis in 68 (67.3%) cases whilst it preceded psoriasis in the remaining 33 (32.7%) cases. In cases where BP followed the diagnosis of psoriasis, the mean (SD) latency between the diseases was 11.8 (10.7) years, and the median (range) latency was 10.1 (0.1–45.0) years. In cases where BP preceded the diagnosis of psoriasis, an average (SD) and median (range) interval of 3.8 (4.0) and 2.0 (0.2–14.9) years, respectively, were observed.

### The Epidemiological and Clinical Features of Patients With BP and Psoriasis Relative to The Remaining Patients With BP

The last endpoint of the current study was to delineate whether patients with a dual diagnosis of BP and psoriasis possess distinct characteristics relative to the remaining patients with BP (Table 5). Patients with coexistent BP and psoriasis were significantly younger at the onset of BP [74.4(11.9) vs. 76.8(14.4); (*P* = 0.049), were more likely to be male (53.5 vs. 42.2%; *P* = 0.023), higher prevalence rate of hypertension (84.2 vs. 74.9%; *P* = 0.033), and smoking (53.5 vs. 28.6%; *P* < 0.001). In addition, higher proportion of patients with BP and psoriasis were managed by prolonged systemic (74.3 vs. 64.8%; *P* = 0.048) and topical (99.0 vs. 94.0%; *P* = 0.035) corticosteroids whereas lower proportion received adjuvant agents (69.3 vs. 80.3%; *P* = 0.006).

**Table 5:** Comparison between patients with coexistent bullous pemphigoid and psoriasis relative to the remaining patients with bullous pemphigoid.

	**Coexistent BP**	**BP without**	***P*-value**
	**and psoriasis**	**psoriasis**	
	**(*n =* 101)**	**(*n =* 3,823)**	
Age at the onset of BP, years; mean (SD)	74.4 (11.9)	76.8 (14.4)	**0.049**
Male sex, *n* (%)	54 (53.5%)	1,613 (42.2%)	**0.023**
Arab ethnicity, *n* (%)	6 (5.9%)	165 (4.3%)	0.436
Body mass index; Kg/m2, mean (SD)	28.6 (7.1)	27.9 (6.1)	0.257
Diabetes mellitus, *n* (%)	40 (39.6%)	1,584 (41.4%)	0.717
Hyperlipidemia, *n* (%)	80 (79.2%)	2,936 (76.8%)	0.572
Smoking, *n* (%)	54 (53.5%)	1,094 (28.6%)	**<0.001**
Hypertension	85 (84.2%)	2,864 (74.9%)	**0.033**
Charlson comorbidity score; mean (SD)	3.5 (2.5)	3.4 (2.4)	0.692
Long-term systemic corticosteroids, *n* (%)^a^	75 (74.3%)	2,477 (64.8%)	**0.048**
Long-term topical corticosteroids, *n* (%)^b^	100 (99.0%)	3,592 (94.0%)	**0.035**
Adjuvant immunosuppressive or immunomodulatory agents^c^	70 (69.3%)	2,267 (80.3%)	**0.006**
DPP4i-associated BP, *n* (%)	11 (10.9%)	285 (10.1%)	0.793
PD-1/PDL-1- associated BP, *n* (%)	0 (0.0%)5	6 (0.2%)	0.653
Exposure to beta-blockers	71 (70.3%)	2,615 (68.4%)	0.686
Exposure to calcium channel blockers	71 (70.3%)	2,486 (65.0%)	0.273

*n, number; SD, standard deviation*.

a *Patients managed by systemic corticosteroids for more than 6 months*.

b *Patients managed by topical corticosteroids for more than 6 months*.

c *Patients managed by one of the following agents: azathioprine, mycophenolate mofetil, methotrexate, cyclophosphamide, dapsone, doxycycline, rituximab, plasmapheresis, intravenous immunoglobulins*.

***Bold:** significant values*.

No significant difference was disclosed with respect to ethnicity, average BMI, the prevalence of diabetes mellitus, dyslipidemia, average Charlson comorbidity score, the prevalence of the recently reported dipeptidyl peptidase 4 inhibitors (DPP4i)-associated BP and PD-1/PDL-1-associated BP, and the frequency of exposure to the main anti-hypertensive drug classes (Table 5).

## Discussion

The current large-scale population-based retrospective cohort study revealed that patients with BP are at a 2.6-fold increased risk of developing psoriasis. Additionally, individuals with a preceding history of psoriasis were 1.5-times as likely to have BP as those without psoriasis. These estimates remained robust after accounting for potential confounders. Compared to patients with isolated BP, those with coexistent BP and psoriasis were younger at the onset of BP, had a higher prevalence of smoking and hypertension, and were more frequently managed by prolonged systemic and topical corticosteroids and less frequently by adjuvant agents.

### Comment on Our Findings Relative to The Current Literature About the Topic

The coexistence of psoriasis and AIBD was described as early as 1929 (18). Among these diseases, BP and anti-p200 pemphigoid were most frequently reported to coexist with psoriasis (19). In a case series of 145 Japanese patients with coexistent autoimmune blistering skin diseases and psoriasis, BP (63.4%) and anti-p200 pemphigoid (37.2%) were the leading associated disease (19). A review of literature in 2006 depicted 40 cases of coexisting psoriasis and BP, which have been reported since 1960, with no evident differences between classical psoriasis and psoriasis associated with BP (20). In a recent meta-analysis that synthesized data across four cross-sectional and case-control studies encompassing 4,035 patients with BP and 19,215 controls, Phan et al. (12) calculated a pooled OR of 2.5 (95% CI, 1.4–4.6) for psoriasis in patients with BP. However, the literature lacks a precise estimation of the risk of psoriasis among patients with BP based on a longitudinal follow-up.

Our study indicated that BP is associated with a 2.6-fold increased risk of newly diagnosed psoriasis. Correspondingly, 32.7% of patients with dual diagnoses developed psoriasis after being diagnosed with BP. This observation does not accord with that reported in the Japanese study, where only 2.8% of patients with coexistent AIBD and psoriasis had their psoriasis diagnosed subsequent to AIBD (19). The main interpretation underlying this discrepancy stems from the different settings of the studies. The Japanese study was held in a large referral center specialized in AIBD which collects samples from all over the country (92.4% of their eligible cases were consultations from other centers), rendering it susceptible to selection bias as well as to overlook the diagnosis of psoriasis since these patients were originally followed for AIBD rather than for psoriasis. Our study, on the other hand, encompassed all levels of healthcare services, including outpatient and inpatient settings, and was more likely to detect documentation of psoriasis in outpatient setting even years after the diagnosis of BP.

While females are more predisposed to the majority of autoimmune diseases, we found that the bidirectional association between BP and psoriasis was more prominent among males. A corresponding male predilection was also observed in the meta-analysis of Phan et al. (12), where the pooled prevalence of psoriasis was 1.8-fold higher among male patients with BP as compared to their female counterparts. Further investigation is warranted to reveal the underlying mechanism of this intriguing observation. The increased burden of hypertension among patients with coexistent BP and psoriasis may reflect the higher exposure to systemic corticosteroids in this subgroup. Differential exposure to anti-hypertensive drugs between BP patients with and without psoriasis was refuted.

### Interpretation of The Findings

The pathomechanism underlying this association is largely unknown. However, several hypotheses had been postulated aiming to account for this comorbidity. The common denominator in both diseases is the basement membrane. Previous studies have depicted that laminin 1 and laminin α1 within the basement membrane zone (BMZ) are disrupted in both involved and uninvolved psoriatic lesions (21, 22). Degradation of laminin in psoriasis is accelerated by the overexpression of fibronectin, α5β1 integrin, and plasminogen activators (23). These structural alterations may modify the antigenicity of BMZ and lower the threshold for the generation of anti-BMZ autoantibodies. Another hypothesis refers to the shared role of neutrophils both in BP and psoriasis, given that keratinocytes in both conditions produce neutrophil chemoattractants, and neutrophilic infiltrate is histologically present in both diseases (24). It was found that neutrophils secrete several metalloproteases that may be implicated in degradation of matrix proteins, leading to subsequent exposure of antigenic epitopes from matrix autoantigens forming the BMZ (25).

Cytokines and T-cell polarization might be an additional factor involved in the coexistence of BP and psoriasis. Interleukin (IL)-1 is a pro-inflammatory and immunomodulatory cytokine that plays a central role in both psoriasis and BP. Yano et al. (26) reported that IL-1-regulated genes are associated with proteolysis, signal transduction, adhesion, proliferation, and epidermal differentiation, and concluded that IL-1 is essential for the initiation and formation of psoriatic lesions. Schmidt et al. (27) found that IL-1β levels were significantly elevated in BP blisters relative to those in controls. Ameglio et al. (28) reported that IL-1β levels were increased in blister fluid compared with those in BP sera and that IL-1β levels correlated with the intensity of the disease. Furthermore, Il-1β has been shown to promote skin inflammation in a pre-clinical mouse model of pemphigoid disease (29). Additionally, serum levels of IL-17 in patients with BP were higher than in control individuals, and the percentage of IL-17-positive cells among CD4+ cells in lesional skin of BP was higher than in that of pemphigus foliaceus (30). In parallel, the essential role of T helper type 17 (Th17) cells and IL-17 is indisputable in the pathogenesis of psoriasis. It is noteworthy that no common susceptibility human leukocyte antigen (HLA) alleles have been reported to overlap between these diseases.

### Strengths and Limitations

The current study utilizes a large-scale study population to address two epidemiological figures; the risk of psoriasis among patients with BP and the risk of BP in individuals with a previous history of psoriasis. The study encompasses two designs to answer the two aforementioned questions, and it represents the first cohort study and the largest case-control study to investigate the association between BP and psoriasis. Knowledge about this comorbidity, as well as other comorbidities, is of great importance to determine the optimal therapeutic options (31, 32). The large sample size assures precision of our estimates, and the population-based setting reduces the possibility of substantial selection bias. However, the current study may be hampered with several limitations, like the absence of severity measures of investigated conditions (BPDAI, ABSIS, and PASI), response to treatments, as well as immunoserological and immunopathological variables. The use of routinely collected data interfered with fulfilling the formal diagnostic criteria of the diseases of interest. Nonetheless, the eligibility criteria were strict, and the first sensitivity analysis has supposedly substantiated the validity of BP diagnosis. We were not able to retrieve data regarding anti-psoriatic therapies, thus leaving the notion that the latter might have mediated the association of BP with psoriasis not refuted. The combination of a diagnosis made by board certified dermatologists and a population based study (in a setting of universal and very accessible healthcare) is unique.

In conclusion, this large-scale population-based study confirms that patients with BP are at a 2.6-fold increased risk of psoriasis. It demonstrates additionally that a history of psoriasis confers a 1.5-fold increase in the odds of developing subsequent BP. Relative to other patients with BP, those with coexistent BP and psoriasis were typified by younger age at the onset of BP, male preponderance, increased burden of smoking and hypertension, and higher administration of prolonged systemic, and topical corticosteroids and lower administration of adjuvant immunomodulatory and immunosuppressive agents. The epidemiological evidence provided herein bears significant implications for physicians managing patients with BP and psoriasis. Further research is required to elucidate the underlying mechanism of this bidirectional association.

## Data Availability Statement

The data analyzed in this study is subject to restrictions. Requests to access these datasets should be directed to dok@clalit.org.i.

## Ethics Statement

The studies involving human participants were reviewed and approved by the institutional review board of Ben-Gurion University 0212-17-COM. Written informed consent for participation was not required for this study in accordance with the national legislation and the institutional requirements.

## Author Contributions

KK and AC: had full access to all of the data in the study and take responsibility for the integrity of the data and the accuracy of the data analysis. KK and RL: study concept and design. KK, YS, and AC: acquisition, analysis, and interpretation of data. KK and GD: drafting of the manuscript. KK, AC, and GD: critical revision of the manuscript for important intellectual content. AC and RL: administrative, technical, or material support. KK and AC: study supervision. All authors contributed to the article and approved the submitted version.

## Conflict of Interest

AC served as an advisor, investigator, or speaker for Abbvie, BI, Dexcel Pharma, Janssen, Novartis, Perrigo, Pfizer, and Rafa. The funders had no role in the design of the study; in the collection, analyses, or interpretation of data; in the writing of the manuscript, or in the decision to publish the results. The remaining authors declare that the research was conducted in the absence of any commercial or financial relationships that could be construed as a potential conflict of interest.

## Abbreviations

BP: bullous pemphigoid
CHS: Clalit Health Services
OR: odds ratio
CI: confidence interval
SD: standard deviation
Th-17: T helper 17
IL: interleukin
DPP4i: Dipeptidyl peptidase 4 inhibitors.
